# Update on Multiple Sclerosis Molecular Biomarkers to Monitor Treatment Effects

**DOI:** 10.3390/jpm12040549

**Published:** 2022-03-31

**Authors:** Viviana Nociti, Marina Romozzi, Massimiliano Mirabella

**Affiliations:** 1 Institute of Neurology, Fondazione Policlinico Universitario ‘Agostino Gemelli’ IRCCS, 00168 Rome, Italy; marinaromozzi@gmail.com (M.R.); massimiliano.mirabella@policlinicogemelli.it (M.M.); 2 Centro di Ricerca Sclerosi Multipla (CERSM), Università Cattolica del Sacro Cuore, 00168 Rome, Italy

**Keywords:** multiple sclerosis, personalized medicine, biomarkers

## Abstract

Multiple sclerosis (MS) is an inflammatory and neurodegenerative disease of the central nervous system characterized by broad inter- and intraindividual heterogeneity. The relapse rate, disability progression, and lesion load assessed through MRI are used to detect disease activity and response to treatment. Although it is possible to standardize these characteristics in larger patient groups, so far, this has been difficult to achieve in individual patients. Easily detectable molecular biomarkers can be powerful tools, permitting a tailored therapy approach for MS patients. However, only a few molecular biomarkers have been routinely used in clinical practice as the validation process, and their transfer into clinical practice takes a long time. This review describes the characteristics of an ideal MS biomarker, the challenges of establishing new biomarkers, and promising molecular biomarkers from blood or CSF samples used to monitor MS treatment effects in clinical practice.

## 1. Introduction

Multiple sclerosis (MS) is a chronic, inflammatory and degenerative disease of the central nervous system (CNS) of unknown aetiology [1]. So far, the pathophysiology of MS seems to be characterized by an aberrant immune activation. This immune dysregulation leads to neuroinflammation in which both immune cells from the periphery and resident cells of the CNS (e.g., microglia and astrocytes) are involved [1]. According to the ‘outside-in’ autoimmune hypothesis, MS is an autoimmune inflammatory disease in which autoreactive T cells and B cells, activated in the periphery, migrate to the CNS and attack various CNS cell types [1]. According to the ‘inside-out’ hypothesis, MS is a primary degenerative disease in which CNS endogenous events may trigger a secondary immune-mediated reaction with the infiltration of autoreactive cell species occurring as a secondary hit [2].

Regardless of the cause of neuroinflammation, well-defined evidence (as well as the successful use of immunomodulatory drugs in reducing disease activity) demonstrates that an uncontrolled inflammatory response in the CNS has a central role in MS [3].

The 2017 revisions of the McDonald criteria permit an early diagnosis of MS, and the growing number of approved disease-modifying therapies (DMTs) allow superior treatment for MS patients by modifying the disease course [4]. Currently, the lesion load in the CNS determined by MRI and clinical characteristics (e.g., relapse rate and disability progression) plays the most important role in treatment choice [5]. However, MS is characterized by significant heterogeneity in radiological and histopathological changes, in clinical features and progression, as well as in therapy response [6]. Therefore, it is very important to define specific features of the disease that facilitate the diagnosis, prognosis, and therapeutic response in every single patient.

Molecular biomarkers are easily quantifiable and can complement MRI and clinical characteristics [7]. The importance of molecular biomarkers from the blood and cerebrospinal fluid (CSF) has been increasingly recognized in recent years; however, since their validation is a lengthy process, at present, it is still difficult to use them routinely in clinical practice [8]. However, the number of potential biomarkers at different stages of testing is promising.

Presently, there are several candidate molecular biomarkers in MS, including diagnostic, prognostic/predictive, disease activity, and treatment–response biomarkers [9].

In the era of emerging therapies for MS, treatment-response biomarkers may permit personalized treatment with DMTs by differentiating treatment responders and non-responders and monitoring side effects. Furthermore, treatment-response biomarkers can be specific to a certain therapy, enabling a tailored therapy approach [8].

This review describes the characteristics of an ideal MS biomarker, the challenges of establishing new biomarkers, and promising molecular biomarkers from blood or CSF samples to monitor MS treatment effects in clinical practice.

## 2. Methods

In order to capture all the relevant articles on treatment-response biomarkers and MS, PubMed was searched using the terms ‘multiple sclerosis’ and ‘treatment-response biomarkers’. The bibliographies of all relevant papers and reviews were hand-searched for additional articles. The data were extracted into spreadsheets. The research was updated until November 2021. All the references were chosen from peer-reviewed journals.

## 3. Precision Medicine in Multiple Sclerosis

### 3.1. Definition and Categorization of Biomarkers

A biomarker is defined as ‘any substance, structure, or process that can be objectively measured in the body or its products and influences or predicts the incidence of outcome or disease’ [10], or pharmacological reactions to therapy [11].

Biomarkers can be of different types: molecular, histological, radiological, or physiological [12]. Biomarkers can be alsoclassified according to their function as susceptibility, diagnostic, prognostic, disease activity and treatment response [13].

Ideal biomarkers should have several common features. An ideal biomarker should differentiate abnormal biological processes from normal processes and should be accessible, non-invasive, cost-effective, reliable, readily available, and easily measured. They should have high specificity (the ability of a test to correctly identify people without the disease) and sensitivity (the ability of a test to designate an individual with the disease as positive). Biomarker levels should be directly linked to a modification in the clinical state, should change promptly in response to therapy, and should be related to the pathophysiological mechanisms occurring in the disease [13] (Table 1).

### 3.2. Biomarkers in Multiple Sclerosis

The study of biomarkers in MS represents an emerging field of research. Currently, candidate molecular biomarkers in MS encompass biomarkers able to predict the onset of the disease, the disease activity, the progression, and the treatment response [9].

**Figure 1:**
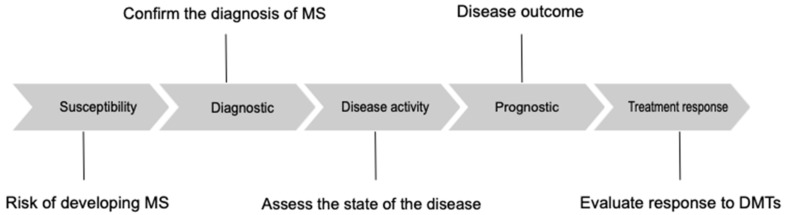
Different types of biomarkers in multiple sclerosis. DMTs, disease-modifying therapies.

Susceptibility biomarkers should identify individuals at risk of developing MS [14]; diagnostic biomarkers should confirm the diagnosis of MS, differentiating patients with MS from patients affected by other diseases. Monitoring biomarkers permit assessment of the state of the disease, detecting active forms of MS and providing an indirect evaluation of treatment response [9]. Prognostic biomarkers provide insight into the overall disease outcome [9]. Lastly, biomarkers of treatment response may permit the treatment of MS patients with tailored therapy (Figure 1) [9].

At present, the relapse rate, disability progression, and lesion load, assessed through MRI, are used to detect disease activity and response to treatment. However, although it is possible to standardize these characteristics in larger patient groups, this has proven difficult to achieve in individual patients [15]. Hence, there is an unmet need for specific, sensitive, and cost-effective biomarkers that are essential to assess therapeutic efficacy and, therefore, to personalize treatment choices in patients with MS [8].

Treatment-response biomarkers in patients with MS may permit personalized treatment with DMTs by selecting patients who are likely to respond to a specific treatment and identifying patients at risk of treatment failure. Their identification and application may also decrease potential adverse effects. Pharmacokinetic/pharmacodynamic and safety biomarkers are part of the aforementioned group and can be used to select and modify the dosage of DMTs [8].

## 4. Treatment-Response Biomarkers in Multiple Sclerosis

Several DMTs for MS have emerged in the last decade with different mechanisms of action.

To date, a number of blood and CSF candidate biomarkers have been proposed to monitor the response to treatments. Oligoclonal bands, circulating antibodies that neutralize against IFNβ, and natalizumab are currently in clinical use. The clinical application of several other markers is the subject of research.

In the paradigm of precision medicine, it is important to understand factors that can determine the therapeutic response and monitor its effectiveness over time. Furthermore, these biomarkers may allow objective assessment of the effectiveness of therapies, even in the absence of clinical deterioration.

### 4.1. Oligoclonal Bands

Oligoclonal bands (OCBs) are immunoglobulin G (IgG) or immunoglobulin M (IgM) class antibodies that are synthesized intrathecally by plasma cells [16]. The presence of at least two bands of IgG within the CSF, but not in the serum, is a strong indication of inflammation in the CNS.

CSF IgG OCBs are found in approximately 90% of patients with MS and 70% of patients with CIS [17]. Furthermore, OCB positivity has a prognostic role of conversion from CIS and radiologically isolated syndrome (RIS) to MS [17,18] and the accumulation of disability [17].

OCBs increase the sensitivity of the diagnosis of RRMS in patients with a first clinical event, suggesting multiple sclerosis and criteria for ‘dissemination in space’ (DIS) [4].

However, OCBs can be found in a significant proportion of patients with other inflammatory neurological disorders and in patients with other neurological diseases [19].

Currently, OCBs represent a validated and clinically applied biomarker with diagnostic and prognostic relevance [9].

Concerning its use as a biomarker of therapeutic response, some DMTs proved to affect intrathecal synthesis with the most consistent data regarding natalizumab, a humanized monoclonal antibody targeting the α4 component of the α4β1 integrin [20,21].

Villar and colleagues demonstrated that a complete therapeutic response to natalizumab in a subset of patients with aggressive MS was associated with a decrease in CSF IgM and, to a lesser degree, in IgG synthesis [20]. In a small series of patients treated with natalizumab, the treatment reduced the CSF OCBs to undetectable levels [22].

In a study performed by Mancuso and colleagues, complete or partial disappearance of CSF OCBs was detected in a large percentage of patients; however, no difference in OCB changes was found when comparing responders to patients with clinical relapses or MRI activity [21].

In a study of 29 treatment-naive RRMS patients receiving cladribine, the disappearance of OCBs after cladribine treatment was associated with a milder disability after 10 years of follow-up [23].

Another study proposed that the absence of OCBs represents favourable prognostic factors influencing the clinical response to interferon-β (IFNβ) and the clinical outcome of IFNβ-treated patients, although the authors could not draw definitive conclusions [24].

However, whether suppression of intrathecal humoral response may represent a marker of treatment response is still a matter of debate [23].

### 4.2. C-X-C Motif Chemokine 13

C-X-C motif chemokine 13 (CXCL13) is a crucial homeostatic chemokine expressed in lymphoid organs, and it is essential for the recruitment and compartmentalization of lymphocytes. The main sources of CXCL13 in lymph nodes are stromal cells and follicular dendritic cells; however, CXCL13 is not restricted to the development and maintenance of lymphoid tissues. It is also involved in mechanisms of chronic inflammation through the formation of tertiary lymphoid structures [25,26]. In MS, CXCL13 regulates homing of B cells and subsets of T cells to inflammatory foci in CNS by interacting with the CXCR5 receptor. The levels of CXCL13 are elevated in the CSF of patients with MS compared to healthy controls [27], as well as in other neuroinflammatory diseases [28]. CXCL13 may be considered a CSF biomarker of intrathecal B cell response, as its levels correlate with the count of B cells, the IgG index, and the presence and OCBs in the CSF [29].

In addition, CXCL13 represents a prognostic marker in patients with CIS, predicting conversion to MS [30]. Higher CSF CXCL13 levels are also associated with disease activity and with a more severe disease course [31]. Similarly, the serum levels of CXCL13 are correlated with active MS [32].

CXCL13 levels can be elevated even in neuroborreliosis and other infectious diseases of the CNS [33]. Thus, its levels indicate a strong humoral immune response in the CNS rather than a specific disease [34].

Nevertheless, CXCL13 may be a useful biomarker for treatment response in MS. Sellebjerg and colleagues demonstrated that in patients treated with natalizumab, the CSF concentration of CXCL13 decreased after treatment with natalizumab [27].

Novakova and colleagues found reduced CSF levels of CXCL13 in patients switching from first-line DMTs to fingolimod [35].

However, the linkage between CXCL13 levels and humoral response in CNS implies that this biomarker could be useful in monitoring the efficacy of B-cell-depleting therapies. After treatment with rituximab, a B-cell-depleting anti-CD20 monoclonal antibody, CXCL13 levels decreased in the CSF. Hence, it has been postulated that elevated CSF CXCL13 levels might predict the response to B-cell-depleting agents [36]. Furthermore, patients with RRMS treated with rituximab had a reduction in CSF T cells correlated with the proportional decrease in CXCL13 levels. This led to speculation that B cell depletion leads to secondary T cell depletion through the reduced production of CXCL13 [37]. At present, CXCL13 is not used in clinical practice.

### 4.3. Osteopontin

Osteopontin is a pro-inflammatory cytokine secreted by activated immune cells and is involved in numerous physiological and pathological processes, such as bone remodelling and immune response [38]. Osteopontin induces the production of interleukin (IL)-12, IL-17, and interferon- γ (INF-γ) [39]. It is also involved in MS pathogenesis and diminishes levels of IL-10, a neuroprotective cytokine in MS [39].

Plasma and CSF osteopontin levels are elevated in patients with MS when compared with healthy controls [40,41,42,43]. CSF osteopontin levels are significantly higher in RRMS during relapse (indicating disease activity) and in PPMS, where higher levels correlated significantly with the degree of disability [44,45]. Plasma osteopontin levels are also higher during relapses [46] and in patients with SPMS when compared with RRMS patients and healthy controls [40,47].

Anyway, elevated levels of plasma and CSF osteopontin may be found in other neurological diseases; thus, it is not a disease-specific marker [40,42].

Regarding osteopontin’s potential role as a treatment-response biomarker, therapy with natalizumab or glatiramer acetate compared to no therapy was associated with lower circulating levels of osteopontin, while treatment with interferon (INF) did not lead to a significant decrease in osteopontin levels [47]. Similarly, decreased CSF levels of osteopontin during treatment with natalizumab in patients with MS have been demonstrated [48].

### 4.4. Neutralizing Antibodies

Neutralizing antibodies (NAbs) can be generated in response to the administration of protein-based drugs and can interfere with the mechanism of action of the drug. The development of NAbs has been observed after exposure to DMTs in MS. At present, NAbs represent a clinically useful biomarker to guide therapeutic decisions.

#### 4.4.1. Neutralizing Antibodies against Interferon-β

Interferon-β (IFNβ) is an established first-line treatment in RRMS. About 40% of patients treated with IFNβ are categorized as non-responders [49]. Up to 40% of patients treated with IFNβ generate serum NAbs, usually during the first year of treatment [50]. The development of NAbs varies depending on the type of IFNβ and its route of administration, with higher and lower levels being associated with the subcutaneous formulation of IFNβ-1b and the intramuscular formulation of IFNβ-1a, respectively [51,52].

Neutralizing antibodies reduce the therapeutic efficacy of IFNβ and negatively impact the clinical course, radiological activity, and disability progression [53,54,55].

The European Guidelines recommend the suspension of IFNß therapy in patients with high titers of NAbs that are persistent at repeated measurements [53,56].

The detection of NAbs against IFNβ can be indicative of a poor therapeutic response. NAb testing, together with clinical and radiological parameters, can help the clinician make therapeutic decisions [56].

#### 4.4.2. Neutralizing Antibodies against Natalizumab

Approximately 9% of MS patients treated with natalizumab develop serum NAbs against the drug, of which 3% of patients have a transient positivity, and 6% of patients have a persistent positivity defined by two consecutive detections [57,58]. These antibodies are most often found within the first six months of treatment [57]. A high titre of baseline NAbs against natalizumab accurately predicts persistency [59].

Neutralizing antibodies against natalizumab are associated with reduced serum levels of the drug and, consequently, with a reduction in the therapeutic efficacy of natalizumab, determined by comparing the relapse rate, radiological activity, and disability progression to antibody-negative patients [57]. Furthermore, persistently antibody-positive patients had a higher rate of infusion-related adverse events [57].

Currently, there is no consensus on testing for NAbs against natalizumab [9]. These antibodies may represent a valuable biomarker to monitor natalizumab efficacy and to tailor treatment for individual patients, particularly for patients with a suboptimal response to the drug during the first year of treatment, drug-related adverse events, or late hypersensitivity reactions [59,60]. Given the concerns surrounding the risks of natalizumab therapy [61], in cases of NAb detection and persistency, a therapeutic switch may be considered. However, as the discontinuation of natalizumab may be associated with disease rebound [62], this must be considered carefully.

### 4.5. Myxovirus Resistance Protein A

Myxovirus resistance protein A (MxA) is an antiviral protein belonging to the family of GTPases [63]. MxA is induced by IFNβ, and therefore, it indicates the biological activity of IFNβ [64]. The detection of MxA mRNA in the peripheral blood mononuclear cells has been revealed to detect the patients in whom IFNβ does not activate the corresponding receptor [56,65]. Thus, low MxA levels correspond to low IFNβ bioavailability and allow for the identification of biological non-responders.

Furthermore, if neutralizing antibodies against IFNβ with a low to medium titre are detected in an MS patient, the MxA quantification can provide supplementary information, and a change of treatment can be considered [56].

### 4.6. Neurofilaments

Neurofilaments (NFs) are axonal cytoskeletal proteins composed of a light chain (NFL), a medium chain (NFM), and a heavy chain (NFH), according to molecular weight [66]. Neurofilaments have a primary structural role in axons and are involved in axonal transport [67].

Axonal injury determines the release of NFs in CSF and serum, reflecting the extent of the axonal damage in different neurological disorders, including MS [68].

Cerebrospinal fluid levels of NFL are elevated in patients with MS compared with healthy controls, with the highest levels emerging during relapses, reflecting the acute, inflammatory-mediated axonal damage [69]. Increased CSF NFL levels were found to be a risk factor for the conversion from CIS to a clinically definite MS [70].

Similarly, serum NFL levels are increased in MS patients when compared to healthy controls and positively correlate with MRI activity, relapses, or deterioration of physical disability [71,72,73].

The levels of CSF NFH are higher in all diseases stages and relapses when compared with healthy controls, and worsen with ageing [74]. Patients with higher levels of CSF NFH have a more significant disability progression when compared to MS patients with normal levels [74]. Moreover, CSF NFH levels are early predictors of the development of brain atrophy in patients with MS [75]. However, NFH concentration in CSF seems to reflect irreversible neuroaxonal degeneration [75,76].

Neurofilaments have been shown to be promising markers of disease activity, disease progression, and prognosis; in addition, some studies are investigating their role as biomarkers for the therapeutic response to DMTs.

Varhaug and colleagues demonstrated that serum NFL levels fell after starting treatment with IFNβ-1a [77]. Plasma NFL levels dropped by 34% after 12 months in MS patients treated with fingolimod (switching from injectable therapies) [78].

Disanto et al. found that the serum NFL concentration was lower in patients treated with DMTs than in untreated patients. In RRMS patients, the decrease in serum NFL levels was inversely correlated with the duration of therapy with DMTs [71]. Furthermore, the authors found a strong association between CSF NFL and serum NFL levels [71].

A cohort of MS patients started on natalizumab experienced a three-fold decrease in CSF NFL levels, dropping to levels similar to the healthy controls after 6–12 months [79]. This evidence is consistent with reduced axonal damage in RRMS treated with natalizumab [79].

Conversely, patients treated with IFNβ had significantly higher levels of CSF NFL than those treated with natalizumab [35].

In progressive MS, NFL levels in CSF showed a significant decrease after 12–24 months of treatment with mitoxantrone or rituximab, suggesting that immunosuppressive treatment may reduce axonal damage, especially in patients with disease activity [80].

In patients with RRMS who shifted from first-line injectable DMTs to rituximab, CSF NFL levels decreased significantly 12 months after the therapy change, indicating that treatment with rituximab may be protective against CNS damage [81].

A post hoc analysis of a phase 3 trial on fingolimod (FREEDOMS) demonstrated a reduction in CSF NFL levels after 12 months of treatment [82].

In the ASCLEPIOS trial, a phase 3 trial of ofatumumab (a subcutaneous anti-CD20 monoclonal antibody) versus teriflunomide, serum NFL was included for the first time as a study endpoint [83]. Serum NFL concentration was significantly lower in the ofatumumab group [83].

Together, these data suggest that both CSF and serum NFL analysis are good potential biomarkers for therapeutic response in MS, especially in response to second-line DMTs, to achieve better prevention of axonal injury [81].

Even if novel, highly sensitive analytic methods (e.g., single-molecule array, SIMOA technology) are now accessible for measuring NFL in both CSF and serum, concerns regarding the test’s availability and replicability, the need for age-matched reference ranges for NFL, and its use in clinical settings remain [84].

### 4.7. Chitinase 3-like Protein 1

Chitinase 3-like protein 1 (CHI3L1), also known as YKL-40, is a chitinase-like glycoprotein, mainly expressed by reactive astrocytes and macrophages, and is involved in chronic inflammation [85]. CHI3L1 was detected in macrophages and astrocytes, predominantly in areas of active demyelination; however, its physiological role in the CNS is still unknown [86].

The concentration of CHI3L1 in the CSF is increased in patients with a higher likelihood of conversion from CIS to MS [87,88]. Higher CSF levels of CHI3L1 are detected in patients with CIS, RRMS, PPMS, SPMS, and MS patients experiencing relapses [89,90,91]. CHI3L1 levels in the CSF are also related to the progression of disability [92,93]. Elevated levels of CSF CHI3L1 correlate with the number of gadolinium-enhancing lesions [87,90].

Other studies have demonstrated a variable increase in CHI3L1 depending on the stage of MS; these studies did not find differences in CHI3L1 levels between relapse and remission [89].

Cantò and colleagues measured plasma CHI3L1 levels in a large cohort of MS patients and demonstrated that CHI3L1 levels were significantly increased in patients with progressive forms of MS when compared with RRMS patients and healthy controls [94]. Anyway, there was not a significant difference in CHI3L1 levels between relapse and remission [94]. In addition, CHI3L1 CSF levels were found to be increased in other inflammatory neurological diseases [91].

The role of CHI3L1 as a potential biomarker has not yet been established [95]. Regarding its possible use as a biomarker to track the response to treatments, a tendency towards decreased plasma CHI3L1 levels was seen in patients treated with IFNβ [94].

A study on peripheral blood CHI3L1 concentration demonstrated that CHI3L1 levels were associated with the response to IFNβ but not to glatiramer acetate [96].

Furthermore, CSF levels of CHI3L1 decreased after natalizumab and mitoxantrone treatment [89].

Stoop and colleagues found a significant reduction in CSF CHI3L1 levels in MS patients after 1 year of natalizumab treatment [97].

Another study demonstrated that fingolimod treatment reduced CHI3L1 concentrations in CSF, along with CXCL13 and NFL, in patients with RRMS previously treated with first-line therapies [98].

CHI3L1 may be regarded as a potential biomarker for the treatment response to IFNβ and natalizumab; however, studies on plasma levels of this biomarker are scarce.

## 5. Conclusions

This review provides a comprehensive, thorough, and updated understanding of treatment biomarkers in MS and offers critical analyses of standing works. A limitation of this review was the partial, subjective weighing of the studies chosen for the review.

In previous decades, significant progress has been achieved in our understanding of the pathophysiological and immunological mechanisms of MS. Moreover, novel, highly effective therapies are emerging in the treatment of MS. Clinical and radiological features are currently routinely used to evaluate individual treatment responses of patients with MS. Hence, biomarkers may allow for the reveal of subclinical deterioration and give the opportunity to ‘tailor’ the treatment.

The need to treat patients with MS earlier and to personalize therapy is hampered by a lack of useful biomarkers that can predict treatment outcomes in individual patients. In addition, clinical trials of new MS drugs require biomarkers that can predict response to therapy over time. These biomarkers need to be rapid, cost-effective, non-invasive, easily measured, and uniformly administered across multiple centres. At present, OCBs, neutralizing antibodies against IFNβ, and natalizumab are used in clinical practice. The best-known biomarker for MS at the molecular level is the presence of OCBs. OCBs have diagnostic relevance for MS, and in addition, the presence of OCBs is a prognostic biomarker and a potential marker of treatment response. Similarly, the use of neutralizing antibodies against IFNβ, natalizumab, and MxA is established in clinical practice. Neurofilaments represent a possible valid biomarker, and osteopontin, CHI3L, and CXCL13 are still exploratory biomarkers that seem to be promising candidates for monitoring therapeutic response in MS, but clinical validation is still required (Table 2).

**Table 2:** Validated and promising treatment-response biomarkers and their functions.

Biomarker	Function	References
**OCBs**	IgG or IgM antibodies synthesized intrathecally by plasma cells	[16,17,18,19,20,21,22,23,24]
**CXCL13**	Chemokine expressed in lymphoid organs, essential for the recruitment of lymphocytes	[25,26,27,28,29,30,31,32,33,34,35,36,37]
**Osteopontin**	Pro-inflammatory cytokine secreted by activated immune cells	[38,39,40,41,42,43,44,45,46,47,48]
**NAbs against IFN-ß**	Serum antibodies against IFNβ	[49,50,51,52,53,54,55,56]
**NAbs against natalizumab**	Serum antibodies against natalizumab	[57,58,59,60,61,62,63,64,65,66,67,68,69,70,71,72]
**MxA**	Antiviral protein induced by IFNβ	[56,63,64,65]
**Neurofilaments**	Axonal cytoskeletal proteins	[66,67,68,69,70,71,72,73,74,75,76,77,78,79,80,81,82,83,84]
**CHI3L1**	Chitinase-like glycoprotein, expressed by astrocytes and macrophages	[85,86,87,88,89,90,91,92,93,94,95,96,97,98]

Ig, Immunoglobulin; OCB, Oligoclonal bands; CXCL13, C-X-C motif chemokine 13; NAbs, Neutralizing antibodies; IFNβ, Interferon-β; MxA, Myxovirus resistance protein A; CHI3L1, Chitinase 3-like protein.

Some of these markers (e.g., NFL) are not disease-specific, and efforts should be made to define cut-off levels in relation to sex, age, and other comorbidities, as these factors may cause intra- and interindividual variability. Furthermore, most of the studies were performed on CSF, but there is an urgent need to develop reliable serum biomarkers that are more accessible, non-invasive, and readily available.

Despite numerous advances in recent years, the biggest challenge in MS biomarker research continues to be the lack of reproducibility and sensitivity. Advances in ‘omics’ technologies will open new avenues for biomarkers research. The ‘omics’ approach will change the paradigm of the hypothesis-based single-marker towards a panel of multiple biomarkers that reflect multiple disease mechanisms, profiling the MS endophenotype [99].

The small number of validated biomarkers used in clinical practice to monitor treatment response indicates that personalized medicine for MS is still in its infancy. However, several candidate biomarkers have been studied and need validation, and ‘omics’ technologies are rapidly evolving, providing the basis for future research prospects.

## Figures and Tables

**Table 1:** Advantages and disadvantages of blood and CSF biomarkers. Molecular biomarkers discussed in the review.

	Advantages	Disadvantages	Molecular Biomarkers
**Blood**	Accessible Non-invasive Cost-effective Time-efficient Easily measured Large quantities Acceptability	Does not always reflect changes in CNS Lower concentration of the biomarker Preanalytical bias	NFL CHI3L1 Osteopontin MxA NAbs against natalizumab and INF-ß
**CSF**	Reflects changes in CNS Lower concentration of the biomarker	Invasive Accessibility Small quantities Acceptability Preanalytical bias	NFL CXCL13 CHI3L1 OCBs Osteopontin

CHI3L1, Chitinase 3-like protein; CXCL13, C-X-C motif chemokine 13; OCB, Oligoclonal bands; NAbs, Neutralizing antibodies; IFNβ, Interferon-β; NF, neurofilaments; MxA, Myxovirus resistance protein A; CSF, cerebrospinal fluid.
